# A Facile Strategy to Prepare Dendrimer-stabilized Gold Nanorods with Sub-10-nm Size for Efficient Photothermal Cancer Therapy

**DOI:** 10.1038/srep22764

**Published:** 2016-03-09

**Authors:** Xinyu Wang, Hanling Wang, Yitong Wang, Xiangtong Yu, Sanjun Zhang, Qiang Zhang, Yiyun Cheng

**Affiliations:** 1 Shanghai Key Laboratory of Regulatory Biology, School of Life sciences, East China Normal University, Shanghai, 200241, P.R. China; 2 State Key Laboratory of Precision Spectroscopy, East China Normal University, Shanghai, 200241, P.R. China

## Abstract

Gold (Au) nanoparticles are promising photothermal agents with the potential of clinical translation. However, the safety concerns of Au photothermal agents including the potential toxic compositions such as silver and copper elements in their structures and the relative large size-caused retention and accumulation in the body post-treatment are still questionable. In this article, we successfully synthesized dendrimer-stabilized Au nanorods (DSAuNRs) with pure Au composition and a sub-10-nm size in length, which represented much higher photothermal effect compared with dendrimer-encapsulated Au nanoparticles due to their significantly enhanced absorption in the near-infrared region. Furthermore, glycidol-modified DSAuNRs exhibited the excellent biocompatibility and further showed the high photothermal efficiency of killing cancer cells *in vitro* and retarding tumor growth *in vivo*. The investigation depicted an optimal photothermal agent with the desirable size and safe composition.

Nanomaterial-mediated photothermal therapy is a promising strategy for the cancer treatment. To date, a plenty of photothermal agents including metal nanoparticles^1^,^2^,^3^, carbon-based nanomaterials^4^,^5^, polymeric nanoparticles^6^,^7^ and semiconductor nanoparticles^8^,^9^ have been successfully developed, among of which gold (Au) nanoparticles hold the great promise for the clinical translation due to their bio-inert nature^10^ and the clinical trials of Au nanoparticles serving as drug carriers^11^ or photothermal agents^12^,^13^,^14^ for the cancer treatment. However, several safety issues of Au photothermal agents still need improvement before their clinical applications. Au nanoparticles such as nanoshells, nanorods^15^, nanocages^16^, and nanostars^17^ are actually composed of other metal elements such as silver or copper in their structures, which is essentially used for their shape and/or structure-controlled syntheses, but might cause potential toxicity in the body. Besides, the currently-developed Au photothermal agents have relatively large sizes (e.g. ~100 nm for nanoshells^18^, ~50 nm for nanorods^19^,^20^, and >40 nm for nanocages^16^), while the optimal particle size for hepatic clearance is smaller than ~25 nm^21^,^22^, and that for renal secretion is sub-10 nm^23^,^24^,^25^. Therefore, the relative large size of Au nanoparticles would retard their clearance from the body post-treatment, and consequently increase their risk of toxicity in the body. Although few ultrasmall photothermal agents made up of copper sulfide or palladium are recently developed^8^,^25^,^26^, their potential toxic or chemical active compositions are still debatable.

Dendrimers are a class of macromolecules with well-designed architecture, uniform size and porous interior^27^,^28^,^29^,^30^,^31^, which have been widely used in biomedical applications including drug delivery^32^, gene transfection^33^, diagnosis/imaging^34^ and tissue engineering^35^, and also been explored as a template to synthesize ultrasmall nanoparticles (typically less than 5 nm) with high stability and monodispersity^36^,^37^,^38^. Previous studies reported that dendrimer-encapsulated Au nanoparticles (DEAuNPs) have potential applications in photothermal therapy^39^,^40^,^41^. However, the DEAuNPs only showed a weak photothermal effect with a temperature enhancement around 5 °C. In addition, the utilization of visible light (532 nm) and high power density is not feasible for the *in vivo* photothermal treatment.

In this article, we developed a facile method to prepare dendrimer-stabilized Au nanorods (DSAuNRs) with an ultrasmall size of sub-10-nm in length and a pure Au composition, which showed significantly enhanced absorption in the near-infrared (NIR) region compared with DEAuNPs. We first investigated the synthetic mechanism of DSAuNRs, and then evaluated their photothermal effect and the biocompatibility of glycidol-modified DSAuNRs (G-DSAuNRs), and finally assessed the photothermal efficiency of G-DSAuNRs to kill cancer cells *in vitro* and retard tumor growth *in vivo*.

## Results and Discussion

**Figure 1:**
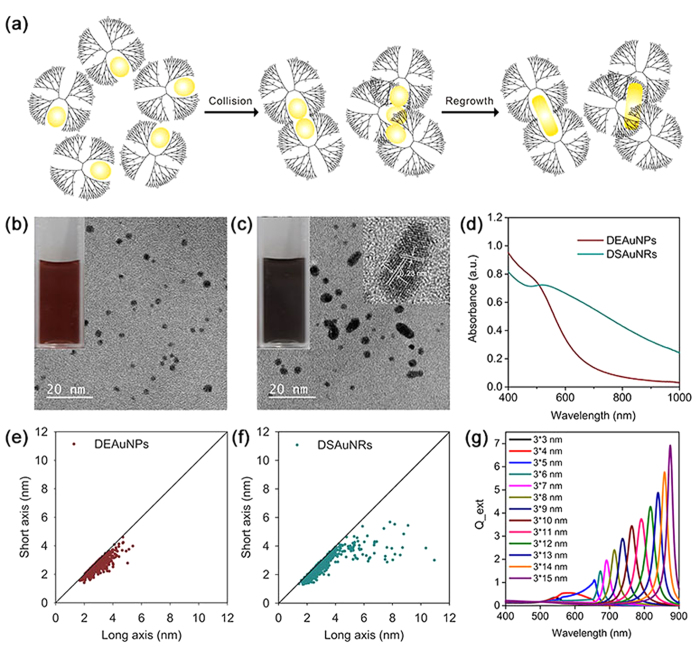
The preparation and characterization of DEAuNPs and DSAuNRs. (**a**) Schematic depicts the possible mechanism for the shape transformation from DEAuNPs to DSAuNRs. (**b,c**) HRTEM images of DEAuNPs and DSAuNRs, respectively. The inset photographs show the bulk solution of DEAuNPs and DSAuNRs, and the inset HRTEM in (**b**) represents a single particle of DSAuNRs. (**d**) The UV-Vis spectra of DEAuNPs and DSAuNRs. (**e,f**) The summarized length (long axis) and width (short axis) distribution of DEAuNPs and DSAuNRs, respectively. (**g**) The simulated extinction efficiency factor (Q_ext) of AuNRs with varied lengths by using DDA method.

**Figure 2:**
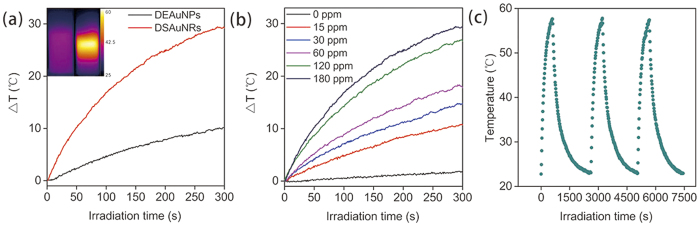
Photothermal effect of DSAuNRs. (**a**) Time-dependent evolution of the temperature for DEAuNPs and DSAuNRs (180 ppm for both) irradiated by NIR laser. Inset represents the thermographs of DEAuNPs (left) and DSAuNRs (right) solution after NIR irradiation. (**b**) Time-dependent temperature changes of DSAuNRs at different Au concentrations irradiated by NIR laser. (**c**) Photothermal stability of DSAuNRs depicted by the cyclic NIR irradiation. All the assays were performed with the individual NIR irradiation at a power density of 2.5 W cm^−2^ for 5 min.

DSAuNRs were prepared via a facile method that DEAuNPs were dialyzed against acidic buffers (Fig. 1a). As shown in Fig. 1b, the original DEAuNPs possessed spherical shape and uniform ultrasmall size of 3.3 ± 0.8 nm, and after dialysis the product was a mix of ultrasmall spherical Au nanoparticles and Au nanorods with different aspect ratios. Since Au nanorods in the product contributed majorly to their photothermal effect (Fig. 2a), the mix was defined as DSAuNRs in this case. The solution color was also changed from wine red for DEAuNPs to black for DSAuNRs (the inset photographs in Fig. 1b,c, respectively), indicating the red-shift of the absorption peaks for DSAuNRs. The statistical analysis suggests that DSAuNRs had a broad aspect ratios ranged from 1 to 4, and had their longest axis to being ~10 nm, while the aspect ratios of DEAuNPs were almost constrained in narrow range of around 1, and their longest axis was smaller than 5 nm (Fig. 1e,f). The inset high-resolution transmission electronic microscopy (HRTEM) image in Fig. 1c shows that DSAuNRs were polycrystalline structure with a lattice fringe of 0.23 nm, which is corresponding to the {111} planes of Au^42^. The UV−Vis spectra show that DEAuNPs had a minimal absorption in the NIR region, while DSAuNRs showed a continuous and significantly boosted absorption from visible to the NIR region (Fig. 1d). The previous studies have demonstrated the localized surface plasmon resonance (LSPR) peaks of Au nanorods were red-shift along with the increase of their aspect ratios^43^. Therefore, the significantly enhanced absorption in the NIR region for DSAuNRs should be attributed to the generated ultrasmall Au nanorods, which is confirmed by the simulated extinction coefficients of ultrasmall Au nanorods with different aspect ratios by using discrete dipole approximation (DDA) method. As shown in Fig. 1g, the extinction of ultrasmall Au nanorods was gradually red-shift as a function of the increased aspect ratios, and the extinction intensity of the single nanorod was also significantly enhanced along with the increase of their aspect ratios, which well explains that few amount of the DSAuNRs with large aspect ratios contributed the considerable NIR absorption for the whole DSAuNRs.

**Figure 3:**
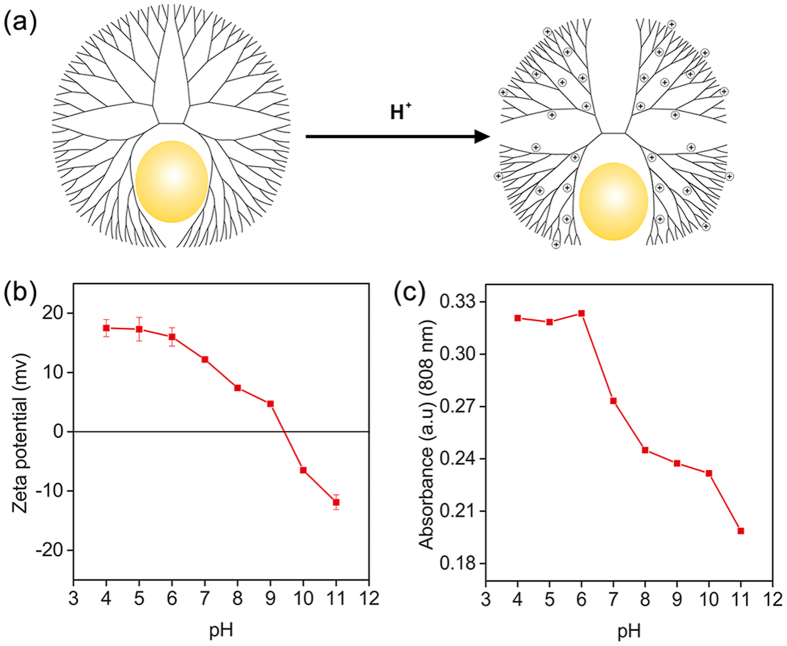
Mechanism of DSAuNRs formation. (**a**) Schematic illustration of protonation and deformation of DEAuNPs in an acidic solution. (**b**) The varied zeta potentials of DEAuNPs in PBS solution at different pH conditions. (**c**) Absorbances of the DSAuNRs at 808 nm prepared by dialysis of DEAuNPs in PBS buffers with different pH values.

To elucidate the possible mechanism for the shape transformation from DEAuNPs into DSAuNRs, DEAuNPs were dialyzed in phosphate buffer solution (PBS) at different pH in a range of 4 to 11, respectively. The initial zeta potential of DEAuNPs in each PBS were measured (Fig. 3b), which showed a pH-dependent decrease with positive charge at pH < 9 and negative charge at pH > 10. The UV-Vis spectra of final products were recorded, in which their absorption at 808 nm also represented a pH-dependent profile (Fig. 3c). The products from DEAuNPs dialyzed in PBS at lower pH conditions had a significantly enhanced NIR absorption, while the ones from DEAuNPs dialyzed in PBS at higher pH conditions had their NIR absorption minimally enhanced. G5-NH_2_ PAMAM dendrimers have large amount of primary and tertiary amines on their repetitive branch units. The protonation of their amines would result in the change of their conformation (Fig. 3a)^44^, leading to exposure of embedded Au nanoparticles within the dendrimer structure. Moreover, the tertiary amine groups within dendrimer pockets may have decreased coordination capability to stabilize Au nanoparticles after protonation. As a result, DEAuNPs became unstable in the acidic solution and underwent a shape transformation into DSAuNRs.

Since DSAuNRs had a considerable absorption in the NIR region, their photothermal effect was further evaluated. First, both of DEAuNPs and DSAuNRs (180 ppm, Au concentration) were irradiated by 808-nm NIR laser at a power density of 2.5 W/cm^2^ for 5 min. As shown in Fig. 2a, the temperatures detected for DSAuNRs were rapidly increased along with the irradiation time and finally achieved an enhancement of around 30 °C, while that for DEAuNPs increased slowly with a small temperature enhancement (~10 °C). Furthermore, DSAuNRs of different concentrations from 15 to 180 ppm were irradiated by NIR laser and their time-elapsed temperature evolution demonstrated that the higher DSAuNRs concentrations, the faster temperature increase (Fig. 2b). The photothermal stability of DSAuNRs was assessed via cyclic-NIR-irradiation assay. As shown in Fig. 2c, there was no decrease of the final temperature for each cyclic irradiation during the measurement period, and no observable changes in the UV-Vis spectra of DSAuNRs before and after NIR irradiation (Figure S1), suggesting DSAuNRs had excellent photothermal stability. Taken together, these results suggest that DSAuNRs were an excellent photothermal agent.

**Figure 4:**
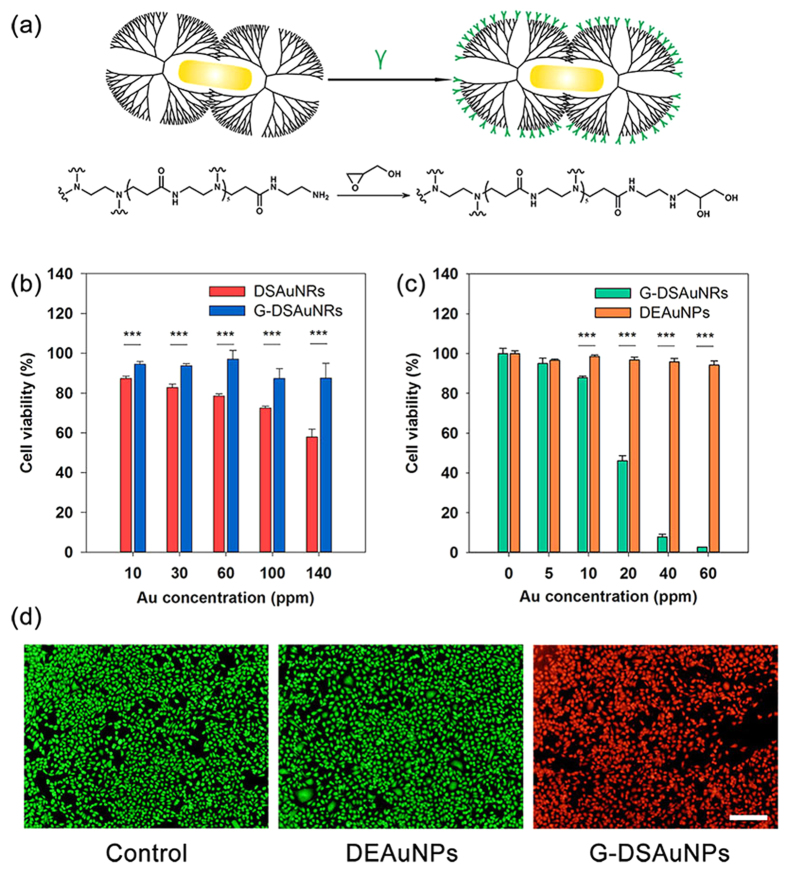
*In vitro* photothermal killing of cancer cells. (**a**) Scheme shows the surface modification of DSAuNRs by glycidol. (**b**) The cytotoxicity of DSAuNRs and G-DSAuNRs on NIH3T3 cells. (**c,d**) The photothermal killing effect of G-DSAuNRs and DEAuNPs on PC-9 cells after NIR irradiation at a power density of 3.6 W cm^−2^ for 5 min revealed by MTT assay (**c**) and AO/EB double-staining assay (**d**). The scale bar in (**d**) is 200 μm. ^***^P < 0.001 was calculated by student’s t-test.

The *in vitro* studies were performed to determine the photothermal killing-cancer efficiency of DSAuNRs. DSAuNRs were first modified with glycidol to remove the primary amine groups on dendrimer surface, which can improve the biocompatibility of cationic dendrimers (Fig. 4a). An average number of 120 glycidol were modified on the surface of each G5 dendrimer, which is characterized by the ^1^H nuclear magnetic resonance (NMR) (Figure S2). The HRTEM image reveals that G-DSAuNRs had the similar size distribution with DSAuNRs (Figure S3), and the dynamic light scattering (DLS) analysis suggests that G-DSAuNRs possessed a hydrodynamic size of 8.95 nm and a zeta potential of 5.36 mV (Figure S4). G-DSAuNRs were incubated with PBS for 3 days, and there was no obvious change over their zeta potential and size (Figure S5). Further, G-DSAuNRs were incubated with fetal bovine serum (FBS) in 50% PBS for 2 hours, and no aggregations or sediments were observed in the suspension (Figure S6). These results suggest that G-DSAuNRs were highly stable in physiological condition. The cytotoxicity of G-DSAuNRs was determined by MTT assay on NIH3T3 cells. As shown in Fig. 4b, The G-DSAuNRs represented an excellent biocompatibility in a broad concentration range of 0–140 ppm, while DSAuNRs were slightly toxic at a concentration above 100 ppm due to the positive charges on the nanoparticle surface, which indicates that the surface modification of glycidol indeed improved the biocompatibility of DSAuNRs. The killing-cancer efficiency of DSAuNRs was further assessed on PC-9 cells, which were treated with different concentration of G-DSAuNRs (0–80 ppm) following with the NIR irradiation at a power density of 3.6 W cm^−2^ for 5 min (Fig. 4c). The relative viability of PC-9 cells was significantly reduced to a small value of less than 5% after the NIR irradiation of the cells treated with G-DSAuNRs at a concentration above 60 ppm, while that of PC-9 cells treated with the same concentration of DEAuNPs plus NIR irradiation at the same powder density showed a minimal viability reduction. Furthermore, the AO/EB staining assay showed that PC-9 cells treated with G-DSAuNRs following with NIR irradiation were nearly 100% killed, while the cells treated with DEAuNPs had no cell death after NIR irradiation. These results suggest that G-DSAuNRs were much more efficient than G-DEAuNPs to kill cancer cells *in vitro*.

**Figure 5:**
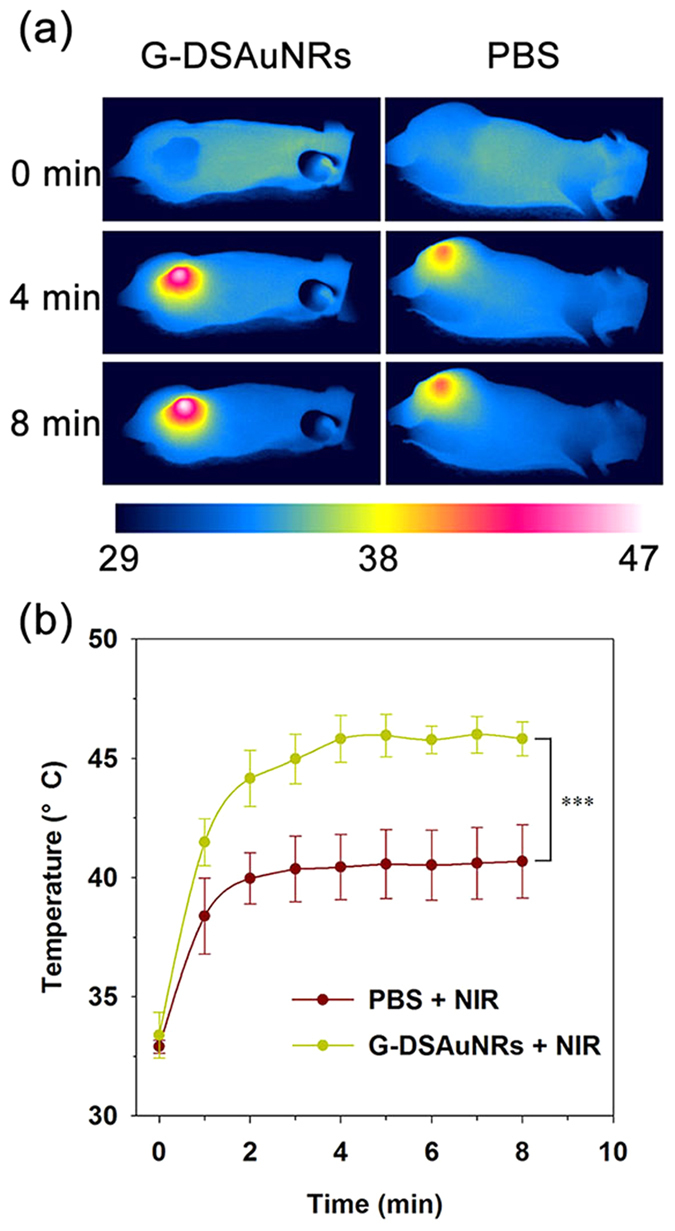
*In vivo* temperature monitoring of animals with different treatments. (**a**) Thermographs of animals treated with G-DSAuNRs (left) and PBS (right) and taken after 8-min NIR irradiation. (**b**) The tumor-site temperature changes of mice treated with G-DSAuNRs and PBS. Animals bearing PC-9 cell xenograft tumors were intravenously administrated with PBS and G-DSAuNRs, respectively, and then were treated with NIR irradiation at a power density of 3.6 W cm^−2^ for 8 min at a time point of 24 h post-injection. ^***^P < 0.001 was calculated by student’s t-test.

**Figure 6:**
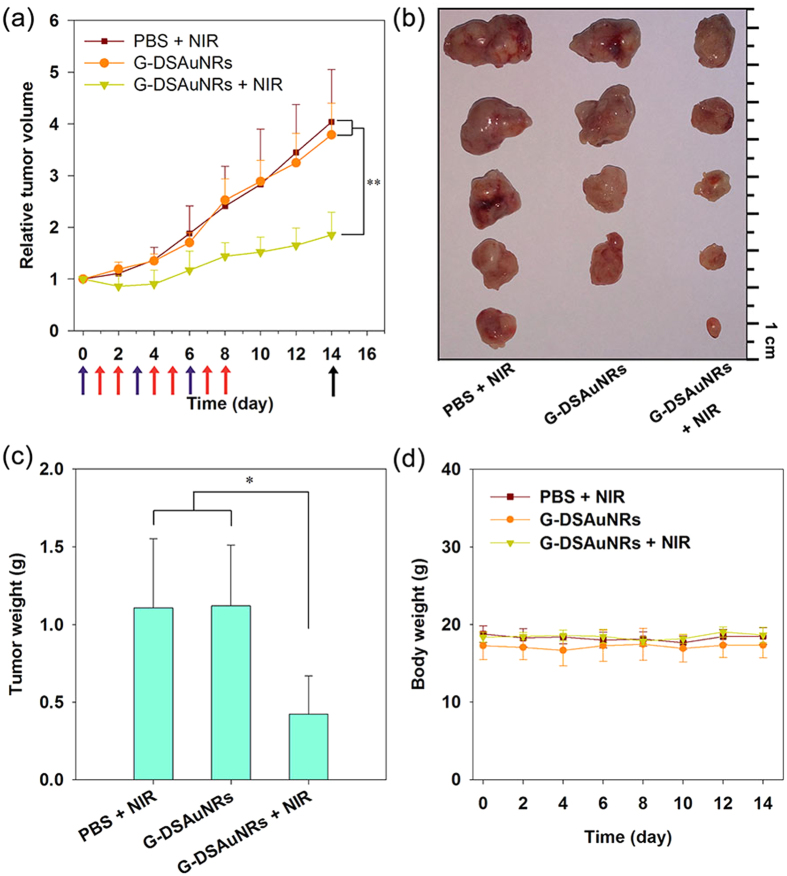
*In vivo* photothermal treatment of tumors by using G-DSAuNRs. (**a**) The evolution of tumor volume for animals with different treatments during the therapeutic period. Red arrows indicate the injection time points, blue ones present the NIR irradiation time points, and black one indicates the sacrificing time point. (**b**) Photographs of the excised tumors from mice sacrificed at the 14^th^ day. (**c**) The average weights of the excised tumors. (**d**) The body weight changes of animals during the therapeutic period. ^*^P < 0.05 and ^**^P < 0.01 were calculated by student’s t-test.

We further conducted the *in vivo* study to determine the photothermal efficacy of G-DSAuNRs for tumor ablation. The nude mice bearing PC-9 xenograft tumors with an average size of 250 mm^3^ were randomly divided into three groups (five mice in each group), and then were intravenously administrated with PBS for one group and G-DSAuNRs for the others two groups. The temperature evolution was recorded for each mouse when irradiated by NIR laser at the time point of 24 h for the first injection. No temperature distinction was observed between the mice treated with PBS and G-DSAuNRs before NIR irradiation, while the tumor-site temperature for the mice treated with G-DSAuNRs was increased fast compared with that for the mice treated with PBS when they were irradiated by NIR laser. The time-elapsed evolution of the tumor-site temperatures revealed that the tumor-site temperature of the mice treated with G-DSAuNRs and then NIR irradiation quickly reached up to an high temperature stage of around 45 °C within 4 min, while that of the mice treated PBS and NIR irradiation finally maintained at a temperature around 40 °C (Fig. 5). These results suggest that G-DSAuNRs were efficiently accumulated in the tumors via the enhanced penetration and retention (EPR) effect to promote the obviously enhanced temperature in tumors upon NIR irradiation. The mice in three groups were administrated with three injections every 3 days and irradiated twice by NIR laser at 24 and 48 h for each injection. During the therapeutic period, the tumor sizes in G-DSAuNRs plus NIR irradiation group were significantly reduced compared with that in the groups of PBS plus NIR irradiation and G-DSAuNRs only (Fig. 6a). The excised tumors and their average weights also showed that the tumor growth in G-DSAuNRs plus NIR irradiation group was significantly retarded (Fig. 6b,c). The mice in the three groups maintained a stable body weights during the therapeutic period, which suggests that G-DSAuNRs had a negligible toxicity (Fig. 6d). The inductively coupled plasma mass spectrometry (ICP-MS) analysis was conducted for the urine and renal tissues that harvested from mice intravenously injected with G-DSAuNRs. The result reveals that there was 6.29 ± 1.91 μg Au /mL in urine and 27.45 ± 5.81 μg Au/g in kidney, indicating G-DSAuNRs could be secreted via renal filtration. Taken together, these results suggest that G-DSAuNRs were an excellent photothermal agent with high photothermal efficacy and minimal toxicity.

In summary, we successfully developed an ultrasmall and pure-Au DSAuNRs for high efficient photothermal therapy. The DSAuNRs were prepared via a facile method that DEAuNPs were dialyzed in acidic buffers for several hours, which represented a significantly higher NIR absorption and consequently more excellent photothermal effect compared with DEAuNPs. The ultrasmall size and the pure-Au composition of DSAuNRs awarded their high safety for *in vivo* photothermal therapy. Furthermore, surface-modified DSAuNRs showed high efficiency to kill cancer cells *in vitro* and retard the tumor growth *in vivo*. This investigation suggests that DSAuNRs is a promising photothermal agent for cancer treatment.

## Methods

### Materials

Chloroauric acid (HAuCl_4_), glycidol and sodium borohydride (NaBH_4_) were purchased from Aladdin Reagent (Shanghai, China). Amine-terminated and generation 5 (G5-NH_2_) PAMAM dendrimer was obtained from Dendritech Inc. (Midland, MI). Hydrochloric acid (HCl) and sodium hydroxide (NaOH) were purchased from Sinopharm Chemical Reagent Co., Ltd. (Shanghai, China). For cell culture, RPMI1640 and DMEM media were purchased from Invitrogen (Carlsbad, CA), and FBS was from Clark Bioscience (Houston, USA). 3-(4, 5-dimethylthiazol-2-yl)-2, 5-diphenyltetrazolium bromide (MTT) was obtained from Sangon Biotech (Shanghai, China). All the reagents were used without further purification.

### Synthesis of DSAuNRs

DSAuNRs were prepared from the shape transformation of DEAuNPs via a dialysis process against acidic buffer. DEAuNPs were first prepared according to the well-developed method by Crooks *et al.*^36^. In a standard synthesis, 1.165 mL of HAuCl_4_ (5 mM) was added into 0.694 mL of G5-NH_2_ (100 μM) in aqueous solution under magnetic stirring at room temperature. 20 min later, 1.665 mL of NaBH_4_ (50 mM, in 0.3 M NaOH) was added dropwise into the reaction solution along with the solution changed from yellow to wine red color, which indicated the generation of DEAuNPs. The reaction solution was stirred for another 30 min to complete the reaction. For the synthesis of DSAuNRs, the aforementioned reaction solution was transferred into a dialysis bag (molecular weight cut off =3500 Da) and dialyzed against PBS (pH = 5). During the dialyzing period, the solution color gradually changed from wine red to black, indicating that DEAuNPs were transformed into DSAuNRs. After that, the reaction solution in the dialysis bag was continuously dialyzed against deionized water for 8 times, and was collected and stored at 4 °C for use. An aliquot of the stock solution was taken to determine the gold concentration by using inductively coupled plasma mass spectrometry (ICP-MS, Agilent 7500 CE, Agilent Technologies, USA).

### Characterization

The high-resolution TEM images were collected using a JEM-2100F microscope (JEOL, Japan) operated at an accelerating voltage of 200 kV. The UV-Vis spectra were recorded by using a UV-Vis spectrometer (Cary60, Agilent Technologies, USA). Zeta potentials were measured by DLS (Zetasizer Nano ZS90, Malvern Instruments Ltd.).

### Synthesis of G-DSAuNRs

In a standard reaction, 3.469 mL of glycidol (50 mM) was added into 12 mL of DSAuNRs (28.90 μM, dendrimer concentration) solution under magnetic stirring, in which the mole ratio between G5-NH_2_ and Glycidol was 1:500. 24 h later, the reaction solution was transferred into a dialysis bag (molecular weight cut off =3500 Da), and then dialyzed against deionized water for 8 times to remove the unreacted glycidol. The final product was collected and stored at 4 °C for use, and an aliquot of the stock solution was taken to determine the gold concentration by using ICP-MS.

### The pH effect over the synthesis of DSAuNRs

To investigate the influence of pH over the transformation of DEAuNPs into DSAuNRs, the fresh-prepared DEAuNPs were dialyzed against PBS solutions at pH values of 4, 5, 6, 7, 8, 9, 10 and 11, respectively for 8 h, and with the change of PBS solution every hour. The final products were analyzed by using UV-Vis spectrometer to determine their absorptions at the wavelength of 808 nm. For the zeta potential measurement, 1 mL of DEAuNPs (3.469 μM, dendrimer concentration) were added in PBS at pH values of 4, 5, 6, 7, 8, 9, 10 and 11, respectively, and were immediately analyzed by DLS. The pH values of PBS buffer were adjusted by the addition of different amounts of HCl (0.3 M) or NaOH (0.3 M) in the solution.

### Photothermal effect of DSAuNRs

To determine the photothermal effect of DSAuNRs, 2 mL of DSAuNRs solutions at different concentrations of 0, 15, 30, 60, 120, 180 ppm (gold concentration) were placed in a plastic cuvette (the cross section dimension is 1 × 1 cm), respectively, and then were irradiated by a NIR laser (808 nm, New Industries Corp., Changchun, China) at a power density of 2.5 W cm^−2^ for 5 min. The fiber-optics probe was ahead of 2 cm above the solution surface with the laser projecting a circular spot of 0.4 cm in diameter on the surface of reaction solution. The photothermal stability of DSAuNRs (180 ppm) was tested with three cycles of NIR irradiation. An infrared thermal camera (Magnity Electronics, China) was employed to record the temperature and thermographs of the reaction solution.

### Cell Culture

Non-small cell lung cancer cell line (PC-9) and mouse embryonic fibroblast cell line (NIH3T3) were obtained from American Type Culture Collection (ATCC). The two cell lines were cultured in 1640 medium supplemented with 10% FBS, penicillin (100 units mL^−1^), and streptomycin (100 mg mL^−1^) at 37 °C under 5% CO_2_.

### Cytotoxicity Assay

NIH3T3 cells were seeded in 96-well plate with a density of 10000 cells per well and incubated overnight at 37 °C. The cells were incubated with DSAuNRs and G-DSAuNRs at different concentrations (10–140 ppm, gold concentration) for 48 h at 37 °C, and then analyzed via the standard MTT assay.

### *In vitro* photothermal efficacy in killing cancer cells

PC-9 cells were seeded in a 96-well plate at a density of 10000 cells per well and incubated overnight at 37 °C. 0.1 mL of G-DSAuNRs at different Au concentrations of 0, 5, 10, 20, 40, 60, 80 ppm were added into the wells, respectively, and then were treated with NIR irradiation at a power density of 3.6 W cm^−2^ for 5 min. After NIR irradiation, the cells were cultured for another 24 h, and then were conducted with the standard MTT assay to determine the cell viability.

### *In vivo* photothermal ablation of tumor

4-week-old male BALB/c nu/nu mice with average weight of 20 g were purchased from Center for Experimental Animals, East China Normal University. The animal experiments were carried out according to the National Institutes of Health guidelines for care and use of laboratory animals and approved by the ethics committee of East China Normal University. For the establishment of PC-9 tumor xenograft model, PC-9 cells (~10^6^ cells, 20 μL) in PBS were subcutaneously injected into the right back of mice. The mice with an average tumor volume of 250 mm^3^ were randomly divided into three groups. The mice in the three groups were intravenously injected with PBS (one group), and G-DSAuNRs (two groups) at a dose of 15 mg Au/kg three times with a time interval of 3 days. The tumor sites of mice in the PBS group and one of the G-DSAuNRs groups were irradiated by NIR laser at a power density of 3.6 W cm^−2^ for 8 min and at a time point of 24 h and 48 h after each injection. The temperature evolution and thermographs were recorded by using an infrared thermal camera when mice were irradiated by NIR laser. The length and width of the tumors and the body weight of mice were measured every two days. The tumor volumes were calculated according to the formula: tumor volume = width^2^ × length × 0.52.

### ICP-MS analysis to determine Au content

Three 4-week-old male BALB/c nu/nu mice were intravenously injected with G-DSAuNRs at a dose of 15 mg/kg, and then were sacrificed 6 hours later to harvest their urine and renal tissues. The samples were then digested by aqua regia and analyzed by using ICP-MS (Agilent 7700X, USA) to determine the concentration of Au in the urine or renal tissues.

## Additional Information

**How to cite this article**: Wang, X. *et al.* A Facile Strategy to Prepare Dendrimer-stabilized Gold Nanorods with Sub-10-nm Size for Efficient Photothermal Cancer Therapy. *Sci. Rep.* **6**, 22764; doi: 10.1038/srep22764 (2016).

## Supplementary Material

Supplementary Information

## References

[b1] Huang X., El-Sayed I. H., Qian W. & El-Sayed M. A. Cancer cell imaging and photothermal therapy in the near-infrared region by using gold nanorods. J. Am. Chem. Soc. 128, 2115–2120 (2006). 16464114 10.1021/ja057254a

[b2] Chen J. *et al.* Immuno gold nanocages with tailored optical properties for targeted photothermal destruction of cancer cells. Nano Lett. 7, 1318–1322 (2007). 17430005 10.1021/nl070345g PMC2504471

[b3] Huang X. *et al.* Freestanding palladium nanosheets with plasmonic and catalytic properties. Nat. Nanotechnol. 6, 28–32 (2011). 21131956 10.1038/nnano.2010.235

[b4] Liu X. *et al.* Optimization of surface chemistry on single-walled carbon nanotubes for *in vivo* photothermal ablation of tumors. Biomaterials 32, 144–151 (2011). 20888630 10.1016/j.biomaterials.2010.08.096

[b5] Robinson J. T. *et al.* Ultrasmall reduced graphene oxide with high near-infrared absorbance for photothermal therapy. J. Am. Chem. Soc. 133, 6825–6831 (2011). 21476500 10.1021/ja2010175

[b6] Liu Y. *et al.* Dopamine-Melanin Colloidal Nanospheres: An Efficient Near-Infrared Photothermal Therapeutic Agent for *In Vivo* Cancer Therapy. Adv. Mater. 25, 1353–1359 (2013). 23280690 10.1002/adma.201204683

[b7] Yang K. *et al.* *In Vitro* and *In Vivo* Near-Infrared Photothermal Therapy of Cancer Using Polypyrrole Organic Nanoparticles. Adv. Mater. 24, 5586–5592 (2012). 22907876 10.1002/adma.201202625

[b8] Zhou M. *et al.* A chelator-free multifunctional [64Cu] CuS nanoparticle platform for simultaneous micro-PET/CT imaging and photothermal ablation therapy. J. Am. Chem. Soc. 132, 15351–15358 (2010). 20942456 10.1021/ja106855m PMC2966020

[b9] Chou S. S. *et al.* Chemically Exfoliated MoS2 as Near-Infrared Photothermal Agents. Angew. Chem., Int. Ed. 125, 4254–4258 (2013). 10.1002/anie.201209229 PMC4193793 23471666

[b10] Cobley C. M., Chen J., Cho E. C., Wang L. V. & Xia Y. Gold nanostructures: a class of multifunctional materials for biomedical applications. Chem. Soc. Rev. 40, 44–56 (2011). 20818451 10.1039/b821763g

[b11] Shao J. *et al.* Photothermal nanodrugs: potential of TNF-gold nanospheres for cancer theranostics. Sci. Rep. 3, 1293 (2013). 23443065 10.1038/srep01293 PMC3582999

[b12] Hirsch L. R. *et al.* Nanoshell-mediated near-infrared thermal therapy of tumors under magnetic resonance guidance. Proc. Natl. Acad. Sci. USA 100, 13549–13554 (2003). 14597719 10.1073/pnas.2232479100 PMC263851

[b13] Rengan A. K., Jagtap M., De A., Banerjee R. & Srivastava R. Multifunctional gold coated thermo-sensitive liposomes for multimodal imaging and photothermal therapy of breast cancer cells. Nanoscale 6, 916–923 (2014). 24281647 10.1039/c3nr04448c

[b14] Rengan A. K. *et al.* *In Vivo* Analysis of Biodegradable Liposome Gold Nanoparticles as Efficient Agents for Photothermal Therapy of Cancer. Nano Lett. 15, 842–848 (2015). 25554860 10.1021/nl5045378

[b15] Huang X., Neretina S. & El-Sayed M. A. Gold nanorods: from synthesis and properties to biological and biomedical applications. Adv. Mater. 21, 4880 (2009). 25378252 10.1002/adma.200802789

[b16] Skrabalak S. E. *et al.* Gold nanocages: synthesis, properties, and applications. Acc. Chem. Res. 41, 1587–1595 (2008). 18570442 10.1021/ar800018v PMC2645935

[b17] He R. *et al.* Facile synthesis of pentacle gold–copper alloy nanocrystals and their plasmonic and catalytic properties. Nat. Comm. 5, 4327 (2014). 10.1038/ncomms5327 PMC4102124 24999674

[b18] Sun Y. & Xia Y. Increased sensitivity of surface plasmon resonance of gold nanoshells compared to that of gold solid colloids in response to environmental changes. Anal. Chem. 74, 5297–5305 (2002). 12403584 10.1021/ac0258352

[b19] Nikoobakht B. & El-Sayed M. A. Preparation and growth mechanism of gold nanorods (NRs) using seed-mediated growth method. Chem. Mater. 15, 1957–1962 (2003).

[b20] Li Z. *et al.* RGD-Conjugated Dendrimer-Modified Gold Nanorods for *in vivo* Tumor Targeting and Photothermal Therapy. Mol. Pharm. 7, 94–104 (2010). 19891496 10.1021/mp9001415

[b21] Longmire M., Choyke P. L. & Kobayashi H. Clearance properties of nano-sized particles and molecules as imaging agents: considerations and caveats. Nanomedicine(Lond) 3, 703–717 (2008). 18817471 10.2217/17435889.3.5.703 PMC3407669

[b22] Wang B., He X., Zhang Z., Zhao Y. & Feng W. Metabolism of nanomaterials *in vivo*: blood circulation and organ clearance. Acc. Chem. Res. 46, 761–769 (2012). 23964655 10.1021/ar2003336

[b23] Choi H. S. *et al.* Renal clearance of quantum dots. Nat. Biotechnol. 25, 1165–1170 (2007). 17891134 10.1038/nbt1340 PMC2702539

[b24] Zhou C. *et al.* Near-Infrared Emitting Radioactive Gold Nanoparticles with Molecular Pharmacokinetics. Angew. Chem., Int. Ed. 124, 10265–10269 (2012). 10.1002/anie.201203031 22961978

[b25] Tang S., Chen M. & Zheng N. Sub-10-nm Pd Nanosheets with Renal Clearance for Efficient Near-Infrared Photothermal Cancer Therapy. Small 10, 3139–3144 (2014). 24729448 10.1002/smll.201303631

[b26] Tian Q. *et al.* Sub-10nm Fe3O4@ Cu2–x S Core–Shell Nanoparticles for Dual-Modal Imaging and Photothermal Therapy. J. Am. Chem. Soc. 135, 8571–8577 (2013). 23687972 10.1021/ja4013497

[b27] Lee C. C., MacKay J. A., Fréchet J. M. J. & Szoka F. C. Designing dendrimers for biological applications. Nat. Biotechnol. 23, 1517–1526 (2005). 16333296 10.1038/nbt1171

[b28] Esfand R. & Tomalia D. A. Poly (amidoamine)(PAMAM) dendrimers: from biomimicry to drug delivery and biomedical applications. Drug Discov. Today 6, 427–436 (2001). 11301287 10.1016/s1359-6446(01)01757-3

[b29] Tomalia D. A. Birth of a new macromolecular architecture: dendrimers as quantized building blocks for nanoscale synthetic polymer chemistry. Prog. Polym. Sci. 30, 294–324 (2005).

[b30] Fréchet J. M. J. & Tomalia D. A. Dendrimers and Other Dendritic Polymers. 2001. *Dendrimers and Dendrons: Concepts, Synthesis, Applications*, 845 (2001).

[b31] Frechet J. Functional polymers and dendrimers: reactivity, molecular architecture, and interfacial energy. Science 263, 1710–1715 (1994). 8134834 10.1126/science.8134834

[b32] Gillies E. R. & Frechet J. M. Dendrimers and dendritic polymers in drug delivery. Drug Discov. Today 10, 35–43 (2005). 15676297 10.1016/S1359-6446(04)03276-3

[b33] Yang J., Zhang Q., Chang H. & Cheng Y. Surface-Engineered Dendrimers in Gene Delivery. Chem. Rev. 115, 5274–5300 (2015). 25944558 10.1021/cr500542t

[b34] Cheng Y., Zhao L., Li Y. & Xu T. Design of biocompatible dendrimers for cancer diagnosis and therapy: current status and future perspectives. Chem. Soc. Rev. 40, 2673–2703 (2011). 21286593 10.1039/c0cs00097c

[b35] Joshi N. & Grinstaff M. Applications of dendrimers in tissue engineering. Current topics in medicinal chemistry 8, 1225–1236 (2008). 18855707 10.2174/156802608785849067

[b36] Crooks R. M., Zhao M., Sun L., Chechik V. & Yeung L. K. Dendrimer-encapsulated metal nanoparticles: synthesis, characterization, and applications to catalysis. Acc. Chem. Res. 34, 181–190 (2001). 11263876 10.1021/ar000110a

[b37] Zhao M. & Crooks R. M. Homogeneous Hydrogenation Catalysis with Monodisperse, Dendrimer-Encapsulated Pd and Pt Nanoparticles. Angew. Chem. Int. Ed. 38, 364–366 (1999). 10.1002/(SICI)1521-3773(19990201)38:3<364::AID-ANIE364>3.0.CO;2-L 29711654

[b38] Scott R. W., Wilson O. M. & Crooks R. M. Synthesis, characterization, and applications of dendrimer-encapsulated nanoparticles. J. Phys. Chem. B. 109, 692–704 (2005). 16866429 10.1021/jp0469665

[b39] Umeda Y., Kojima C., Harada A., Horinaka H. & Kono K. PEG-attached PAMAM dendrimers encapsulating gold nanoparticles: growing gold nanoparticles in the dendrimers for improvement of their photothermal properties. Bioconjug. Chem. 21, 1559–1564 (2010). 20666440 10.1021/bc1001399

[b40] Fukushima D., Sk U. H., Sakamoto Y., Nakase I. & Kojima C. Dual stimuli-sensitive dendrimers: Photothermogenic gold nanoparticle-loaded thermo-responsive elastin-mimetic dendrimers. Colloid. Surface. B 132, 155–160 (2015). 10.1016/j.colsurfb.2015.05.012 26037705

[b41] Haba Y. *et al.* Preparation of poly (ethylene glycol)-modified poly (amido amine) dendrimers encapsulating gold nanoparticles and their heat-generating ability. Langmuir 23, 5243–5246 (2007). 17419657 10.1021/la0700826

[b42] Lee J., Govorov A. O. & Kotov N. A. Nanoparticle assemblies with molecular springs: A nanoscale thermometer. Angew. Chem. Int. Ed. 117, 7605–7608 (2005). 10.1002/anie.200501264 16231378

[b43] Ye X. *et al.* Improved size-tunable synthesis of monodisperse gold nanorods through the use of aromatic additives. ACS Nano 6, 2804–2817 (2012). 22376005 10.1021/nn300315j

[b44] Liu Y., Bryantsev V. S., Diallo M. S. & Goddard Iii W. A. PAMAM dendrimers undergo pH responsive conformational changes without swelling. J. Am. Chem. Soc. 131, 2798–2799 (2009). 19199433 10.1021/ja8100227

