# Supplementary material for: A Facile Strategy to Prepare Dendrimer-stabilized Gold Nanorods with Sub-10-nm Size for Efficient Photothermal Cancer Therapy
Source: Sci Rep. 2016 Mar 9;6:22764. doi: 10.1038/srep22764 (PMC4783698; doi:10.1038/srep22764)
Supplement: Supplementary Information [file srep22764-s1.doc]

Supporting Information

**A Facile Strategy to Prepare Dendrimer-stabilized Gold Nanorods with Sub-10-nm Size for Efficient Photothermal Cancer Therapy**

Xinyu Wang^†§^, Hanling Wang^†§^, Yitong Wang^†^, Xiantong Yu^‡^, Sanjun Zhang^‡^, Qiang Zhang^†^* and Yiyun Cheng^†^*

^†^Shanghai Key Laboratory of Regulatory Biology, School of Life Sciences and ^‡^State Key Laboratory of Precision Spectroscopy, East China Normal University, Shanghai, 200241, P.R. China

Correspondence should be addressed to Y.C. (E-mail: [yycheng@mail.ustc.edu.cn](mailto:yycheng@mail.ustc.edu.cn)); Q. Z. (E-mail: [qzhang@bio.ecnu.edu.cn](mailto:qzhang@bio.ecnu.edu.cn));

^§^These author contributed equally on this manuscript.

**Key words:** dendrimer, gold nanorods, ultrasmall, cancer, photothermal therapy


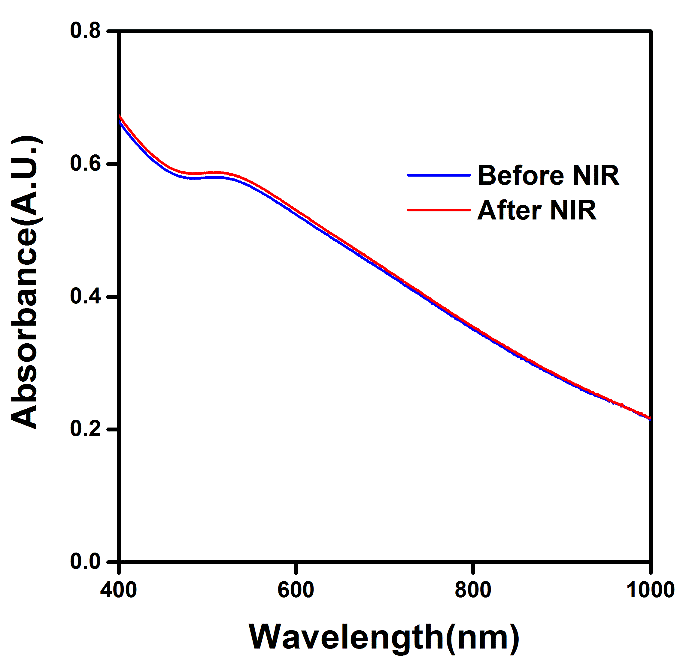


**Figure S1.** UV-Vis spectra of DSAuNRs before and after NIR irradiation.


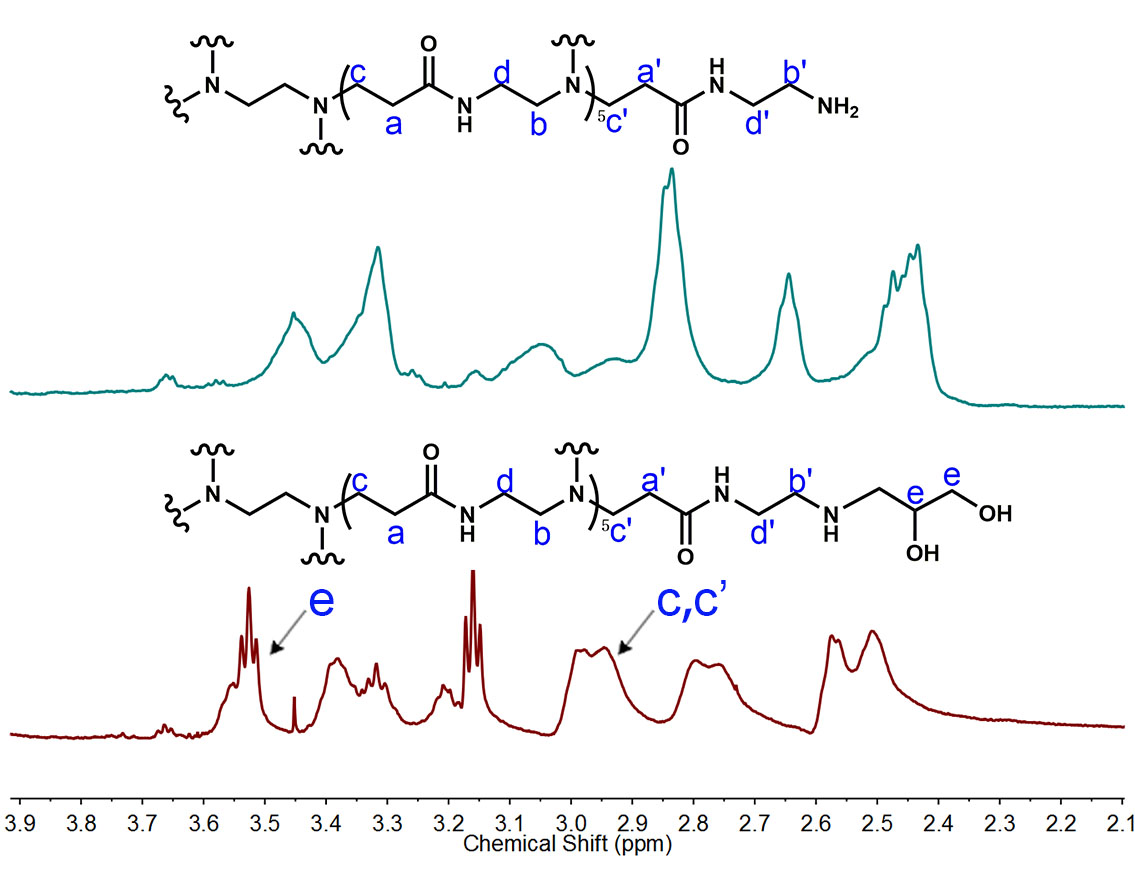


**Figure S2.** ^1^H NMR spectra of DSAuNRs (green) and G-DSAuNRs (red). The average number of glycidol modified on dendrimer was determined by calculating the ratio of peak area of proton e to that of proton c and c’.


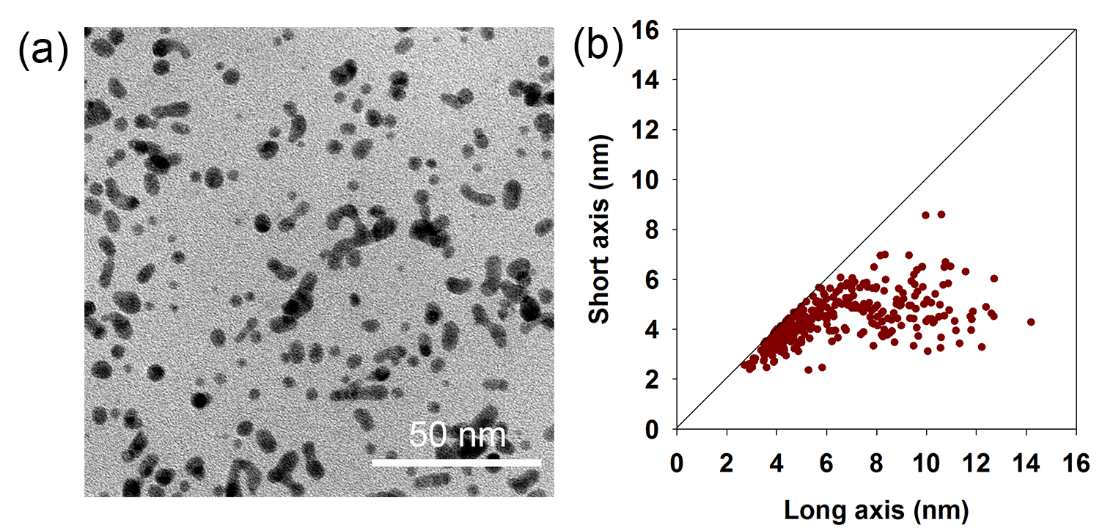


**Figure S3.** (a) HRTEM image of G-DSAuNRs and (b) their corresponding summarized length (long axis) and width (short axis) distribution.


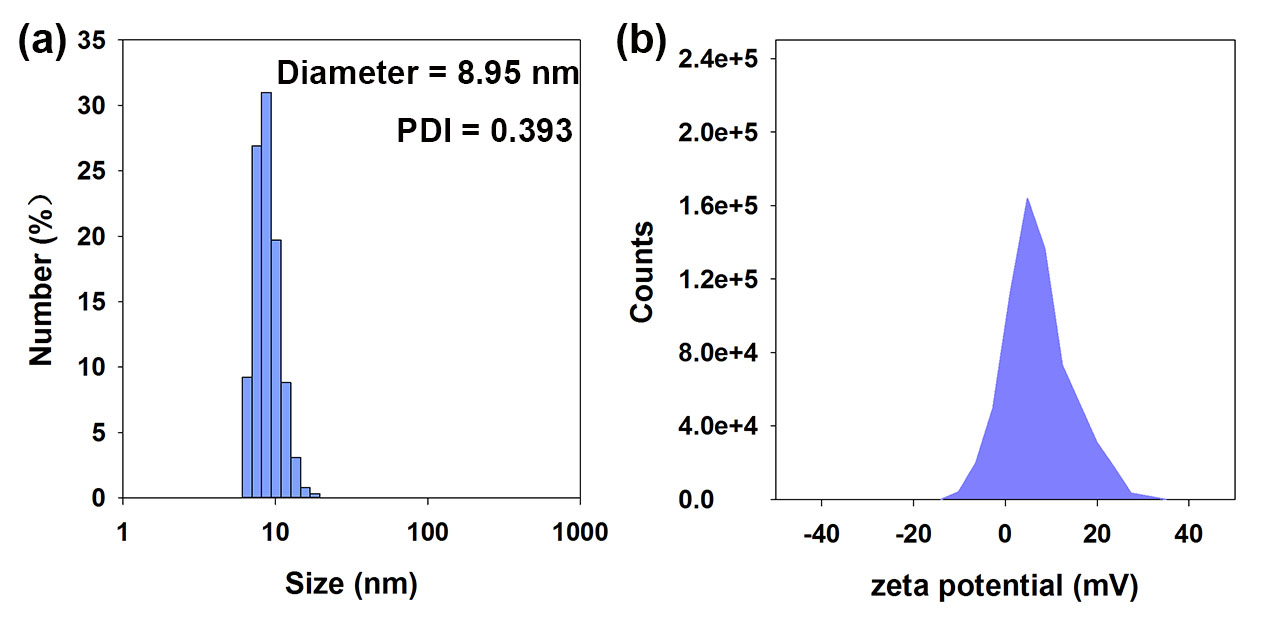


**Figure S4.** (a) Size distribution and (b) zeta potential of G-DSAuNRs was determined by DLS.


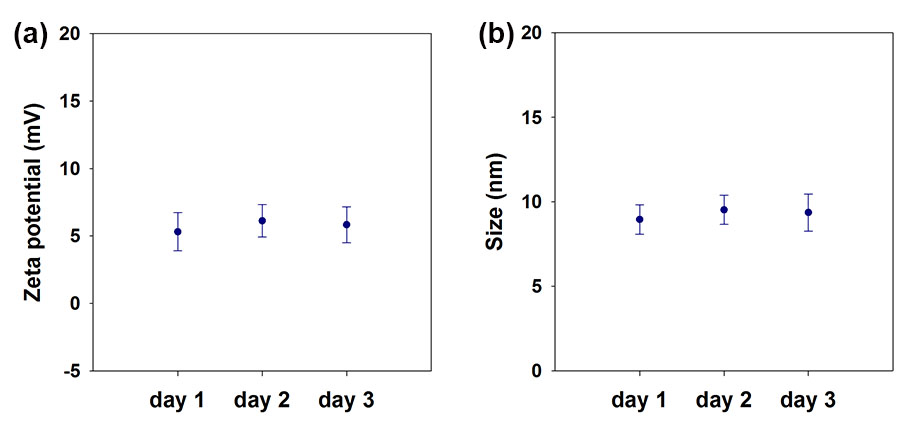


**Figure S5.** Time-elapsed evolution of zeta potential (a) and hydrodynamic size (b) of G-DSAuNRs in PBS for 3 days revealed by DLS.


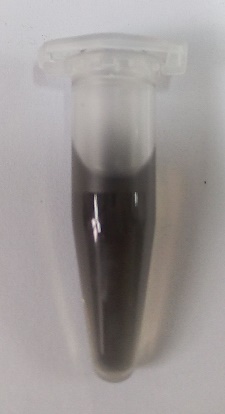


**Figure S6.** Photograph of G-DSAuNRs in FBS (50% in PBS) after 2-h incubation, which indicates that G-DSAuNRs were highly stable in the physiological solution.
